# Research on the Corrosion Resistance of Electrodeposited Ni-SiC Composites Constructed for Steel Storage Tank Application

**DOI:** 10.3390/ma17184616

**Published:** 2024-09-20

**Authors:** Lifu Cui, Xiang Li, Chaoyu Li, Lijie Zhu, Qinggao Zhang, Zheng Li, Haiyu Liu

**Affiliations:** 1 College of Civil Engineering, Dalian Minzu University, Dalian 116600, China; cuilifu@dlnu.edu.cn (L.C.); lixiang@dlnu.edu.cn (X.L.); 202211052060@stu.dlnu.edu.cn (Z.L.); 202211052048@stu.dlnu.edu.cn (H.L.); 2 School of Mechanical and Electrical Engineering, Sanming University, Sanming 365004, China; 3 College of Petroleum Engineering, Northeast Petroleum University, Daqing 163318, China; 218001050083@stu.nepu.edu.cn; 4 College of Civil and Architectural Engineering, Northeast Petroleum University, Daqing 163318, China; zqg@dlnu.edu.cn

**Keywords:** Ni-SiC composites, pulse electrodeposition, structure, corrosion model, anti-corrosion ability

## Abstract

In this paper, the effects of the SiC phase incorporated in Ni substrate deposits on storage tank steel during electrodeposition at different current densities are explored. The microstructure, phase content, and corrosion resistance of the resulting Ni-SiC composites were investigated by scanning electron microscopy (SEM) matched with energy disperse spectroscopy (EDS), X-ray diffraction (XRD), and an electrochemical workstation, respectively. SEM micrographs and EDS results show that at 2.5 A/dm^2^, the composites presented a smooth and compact structure with high SiC content, while at 1.8 or 3.2 A/dm^2^, it became uneven and loose in structure with low SiC content. XRD patterns showed that the nickel grain size of composites firstly increased and then decreased with the growth of the current density. Notably, the Ni-SiC composite produced at 2.5 A/dm^2^ possessed a higher corrosion potential (−0.507 V) and lower corrosion current density (2.439 μA/cm^2^), illustrating that its excellent anti-corrosion ability was superior than that of other two composites. Hence, SiC co-deposited at 2.5 A/dm^2^ conducted as a protective barrier and inhibited the corrosion rate against a corrosion medium of Cl^−^ and SO4^2−^ ions. In addition, the corrosion relationship illustrated that the SiC content of Ni-SiC composite firstly increased and then decreased with the growth of the current density, while the corrosion weight loss of Ni-SiC composites firstly decreased and then increased.

## 1. Introduction

Nowadays, the storage and transportation of crude oil has an important influence on industrial production and economic development [1]. However, crude oil contains abundant water, sulfide, and chloride, leading to the surface of storage tanks being easily corroded by corrosive media. Therefore, various kinds of metallic coating are used to protect the surface of storage tanks and reduce damage from corrosive media [2]. Among these metallic coatings, nickel coatings possess fine processability and stable physical and chemical properties, which can obviously enhance the corrosion resistance of steel matrix [3,4,5]. The common methods used to fabricate nickel-based coatings include electrodeposition [6], laser cladding [7], chemical plating [8], magnetic sputtering [9], and so on. The electrodeposition approach is superior in operation, cost, and efficiency, and is widely used to manufacture nickel-based coatings to be deposited on the surface of steel substrate [10]. However, with continuous extraction from petroleum fields, abundant Cl^−^, SO_4_^2−^, and CO_3_^2−^ ions exist in crude oil. An abundance of the corrosive medium of Cl^−^, SO_4_^2−^, and CO_3_^2−^ ions obviously increases the corrosion ability of crude oil, and obviously decreases the protective effect of the nickel-based coating electrodeposited on the storage tank. As a result, parameter adjustment and various reinforced phases in the methods of operation are usually used to enhance the corrosion resistance of nickel-based composites [11,12,13].

Some researchers studied the effect of operation parameters on the performance of electrodeposited nickel-based composites. For example, Ren et al. [14] discussed the effect of MoS_2_ concentration on the micro-structure and corrosion performance of Ni-WC-MoS_2_ composites manufactured using the electrodeposition method. They proposed that Ni-WC-MoS_2_ composites prepared at an MoS_2_ concentration of 5 g/L had positive corrosion potential, a small corrosion current density, and a low corrosion rate. Jin et al. [15] adopted a jet electrodeposition technique to fabricate Ni-Fe-WC coatings. They concluded that the Ni-Fe-WC coatings manufactured at a low current density or high deposition temperature had a compact, flatted micro-structure and fine strength of coatings. Furthermore, some researchers studied the type of reinforced phase on the performances of electrodeposited nickel-based composites. For instance, Feng et al. [16] manufactured pure Ni coatings and Ni-Al_2_O_3_ composite coatings via a sediment co-deposition approach. They reported that the corrosion resistance and high temperature oxidation resistance of pure nickel improved significantly after the reinforced phase of Al_2_O_3_ was introduced. Wu et al. [17] investigated the effect of Y_2_O_3_ concentration on the micro-structure and anti-corrosion ability of electrodeposited Ni-Mo alloy. They found that the anti-corrosion performance of Ni-Mo composites increased obviously after the incorporation of a Y_2_O_3_ reinforced phase. These results demonstrated that the excellent reinforced phase and appropriate operation parameter contributed to improving the corrosion resistance of Ni-based composites deposited on the surface of substrate. In addition, the reinforced phase of SiC had outstanding stability in the fields of bearings and impellers, which has been proved by Rao et al. [18] and Zhou et al. [19]. However, the literature about electrodeposited Ni-SiC composites applied in steel storage tanks is scarce. Therefore, in this paper, electrodeposited Ni-SiC composites were fabricated and used to enhance the corrosion resistance of storage tanks.

Many relevant studies focus on the anti-corrosion ability of Ni-SiC composites immersed in the corrosion solution, and Cl^−^ ions have been reported by both Chinese and non-Chinese researchers. For example, Ma et al. [20] concluded that the anti-corrosion ability of Ni-SiC composites manufactured at a magnetic intensity of 0.5 T was better than that of those obtained at a magnetic intensity of 0 and 1 T. Rita et al. [21] demonstrated that electrodeposited Ni-SiC composites prepared at pulse frequency of 10 Hz had a higher corrosion protection efficiency than those produced at pulse frequencies of 50 and 100 Hz. However, most researchers have focused on the anti-corrosion performance of electrodeposited Ni-SiC composites immersed in corrosion liquid with Cl^−^ ions, while studies on composites soaked in corrosion medium with Cl^−^ and SO_4_^2−^ ions are few.

Therefore, in this work, the anti-corrosion ability of resulting Ni-SiC composites immersed in corrosion medium with Cl^−^ and SO_4_^2−^ ions was measured by an electrochemical workstation. Also, the surface morphology, phase structure, and content of Ni-SiC composites fabricated at different current densities were investigated by utilizing a scanning electron microscope (SEM), an X-ray diffraction-meter (XRD), and energy disperse spectroscopy (EDS), respectively. In addition, the corrosion relationship between SiC content and corrosion weight loss of Ni-SiC composites produced at different current densities were also analyzed.

## 2. Experiment and Method

### 2.1. Preparation of Ni-SiC Composites 

**Figure 1:**
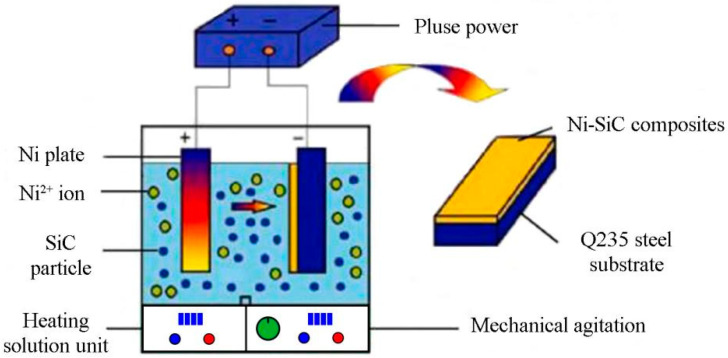
Schematic diagram of facility for depositing Ni-SiC composites.

During the pulse current (PC) electrodeposition process, a nickel plate (60 mm × 30 mm × 5 mm) conducted as an anode, and Q235 steel substrate (40 mm × 13 mm × 2 mm) acted as a cathode, respectively. The nano-sized SiC particles at size of ~20 nm purchased from Guangdong Nanomaterials Co., Ltd. (Dongguan, China) The Q235 steel substrate was mainly composited of Fe (98.39 wt.%), Mn (0.54 wt.%), Cr (0.15 wt.%), C (0.45 wt.%), S (0.02 wt.%), Si (0.27 wt.%), and Cu (0.18 wt.%). Before electrodeposition, the substrates were polished by abrasive papers of 800, 1200, and 1500 grits, respectively. Then, the steel plate was activated in 2 mol/L HCl solution for 30 s and then washed by deionized water. Afterward, the composite electrolyte was prepared mixing SiC nanoparticles, chemical reagent and deionized water. Finally, the nickel plate and Q235 steel substrate were placed in the composite electrolyte and obtained Ni-SiC composites. The schematic diagram of facility for depositing Ni-SiC composites is presented in Figure 1. The specific electrolyte composition and plating parameters for preparing electrodeposited Ni-SiC composites are listed in Table 1. According to the absorption model from Gugliemi, the deposition process divided into two stages: (I) Under the function of electrical field forces generated by pulse power source, the Ni^2+^ ions with SiC nanoparticles adhere to Q235 steel surface; (II) The Ni-SiC composites deposited on the cathodic surface after incorporation of SiC nanoparticles and reduction of Ni^2+^ ions to metallic nickel. The Ni-SiC composites manufactured at current density of 1.8 A/dm^2^, 2.5 A/dm^2^ and 3.2 A/dm^2^ were denoted as NS-1.8, NS-2.5, and NS-3.2 composites, respectively.

### 2.2. Characterization

The surface morphology and corrosion morphology of Ni-SiC composites were investigated by a scanning electron microscope (SEM, S3400, Hitachi, Tokyo, Japan) coupled with an energy dispersive spectrometer (EDS, Hitachi, Tokyo, Japan). The crystalline structure of Ni-SiC composites was determined by a X-ray diffraction (XRD, D-Max/2500, Rigaku, Tokyo, Japan) with Cu Kα radiation at wavelength of 0.15406 nm. The average size of nickel grain in the composites was calculated by utilizing Equation (1):

(1) $$D=\frac{0.89\lambda }{\beta \cos\theta }$$

where D is the average size of nickel-cobalt grain, λ is the wavelength of X-ray, β is the half-width of diffraction peaks, and θ is the Bragg angle, respectively.

The anti-corrosion ability of Ni-SiC composites in the corrosion medium (3.5 wt.% NaCl and 3.5 wt.% Na_2_SO_4_) was measured by a CS350 type electrochemical workstation. The counter electrode, reference electrode and working electrode were Pt electrode, saturated calomel electrode and Ni-SiC composites, respectively. The electrochemical impedance spectroscopy of Ni-SiC composites was measured at a frequency of 10^−2^~10^5^ Hz and amplitude of alternating current (AC) perturbation signal was 10 mV. The contacted area between Ni-SiC composites and corrosion medium was 1 cm^2^. During the corrosion weight loss measurement, the composites were immersed in the same corrosion environment (3.5 wt.% NaCl and 3.5 wt.% Na_2_SO_4_) for 10 days. According to Equation (2), the corrosion weight loss (M) of Ni-SiC composites were assessed.

(2) $$M=M_{0}-M_{1}$$

where M_0_ is the weight of Ni-SiC composites before corrosion test and M_1_ is the weight of Ni-SiCcomposites after corrosion test.

## 3. Results and Discussion

### 3.1. Surface Morphology Investigation

**Figure 2:**
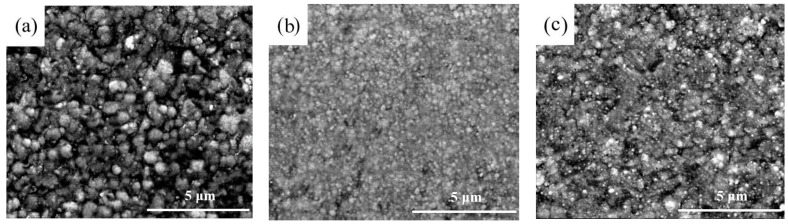
Surface morphologies of Ni-SiC composites obtained at various current densities: (**a**) 1.8 A/dm^2^, (**b**) 2.5 A/dm^2^, and (**c**) 3.2 A/dm^2^.

The SEM graphs of Ni-SiC composites obtained at different current densities are presented in Figure 2. The uneven and loose structure with different size of nickel grains appeared in the surfaces of NS-1.8 composites. By contrast, the flatted and dense structure with fine size of nickel grains generated in the surface of NS-2.5 composites. However, the structure of NS-3.2 composites became loosed and nickel grain size increased. Furthermore, the serious agglomeration phenomenon of SiC nanoparticles existed in the surface of NS-1.8 and NS-3.2 composites, while the uniform distribution of SiC nanoparticles emerged in the surface of NS-2.5 composites.

The reason of this phenomenon could be interpreted that the microstructure of electrodeposited composites was highly related to the current density [22]. The low current density generated a low over-potential and caused the low co-deposition rate of Ni^2+^ ions with SiC nanoparticles [23]. The high current density led to aggravation of concentration polarization and hydrogen evolution reaction, resulting in the co-deposition rate of Ni^2+^ ions with SiC nanoparticles reduced [24]. By contrast, the feasible current density was conducive to enhance the co-deposition rate of Ni^2+^ ions with SiC nanoparticles, which further refined nickel grains size and improved uniform distribution of SiC nanoparticles [25]. Hence, the NS-2.5 composites owned a smooth, compact structure, small nickel grains size, and uniform distribution of SiC nanoparticles.

### 3.2. SiC Content Detection

**Figure 3:**
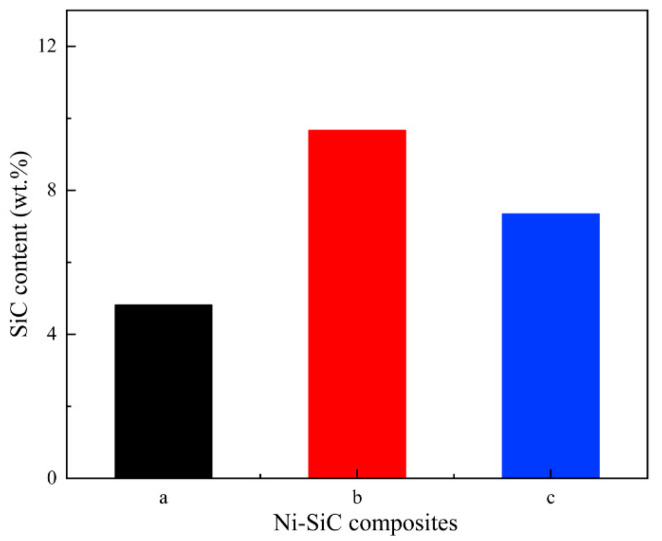
Colummar pictures of SiC content in the Ni-SiC composites manufactured at different current densities: (a) 1.8 A/dm^2^, (b) 2.5 A/dm^2^, and (c) 3.2 A/dm^2^.

The SiC content of Ni-SiC composites deposited at various current densities are shown in Figure 3. The SiC contents of NS-1.8, NS-2.5, and NS-3.2 composites were 4.82 wt.%, 9.67 wt.%, and 7.35 wt.%, respectively. The variation reason of SiC content in the Ni-SiC composites could be illustrated that the appropriate current density contributed to enhance the co-deposition rate of Ni^2+^ ions with SiC nanoparticles and increase the SiC content of Ni-SiC composites. The result was proved by the similar conclusion proposed by the research of Xia et al. [26] about the effect of current density on the TiN content of electrodeposited Ni-TiN composites. Hence, the SiC content of NS-2.5 composites was higher than those of NS-1.8 and NS-3.2 composites.

### 3.3. XRD Patterns Observation

**Figure 4:**
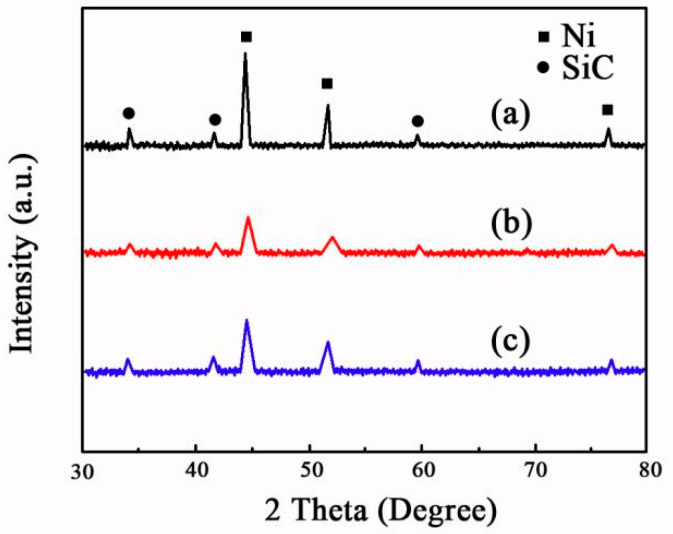
XRD patterns of Ni-SiC composites deposited at different current densities: (a) 1.8 A/dm^2^, (b) 2.5 A/dm^2^, and (c) 3.2 A/dm^2^.

The effect of current density on XRD patterns of Ni-SiC composites are shown in Figure 4. XRD patterns demonstrated that the nickel phase and SiC phase both emerged in three Ni-SiC composites, which represented the SiC nanoparticles and nickel grains were successfully deposited on the substrate surface. The diffraction peak of SiC phase emerged at 34.2°, 41.3°, and 59.7°, corresponding to the planes of (111), (200), and (220), respectively [27]. The diffraction peak of nickel grain emerged at 43.6°, 52.9°, and 75.4°, corresponding to the crystal planes of (111), (200), and (220), respectively [28]. Furthermore, the diffraction peaks location of Ni-SiC composites was not influenced by current density. This result was confirmed to the study of Zhang et al. [29].

**Figure 5:**
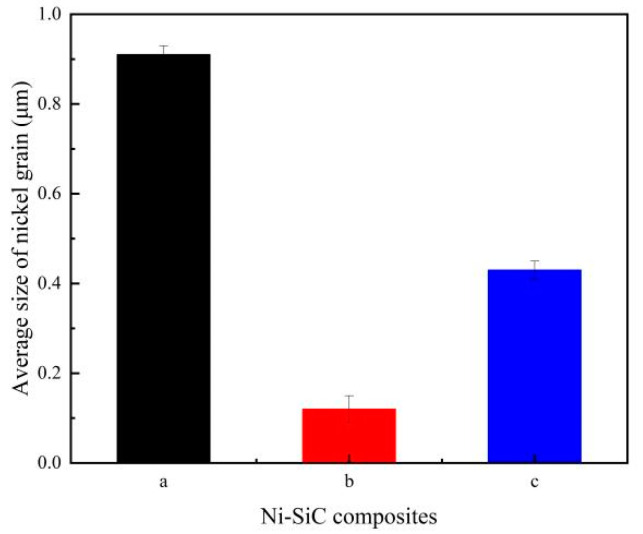
Average size of nickel grain in the Ni-SiC composites prepared at various current densities: (a) 1.8 A/dm^2^, (b) 2.5 A/dm^2^, and (c) 3.2 A/dm^2^.

The average dimension of nickel grain in the Ni-SiC composites are exhibited in Figure 5. The feasible current density contributed to SiC nanoparticles embedded into the Ni matrix. Furthermore, the average size of nickel grain in the Ni-SiC composites decreased firstly and then increased with the growth of current density. According to the calculation of Equation (1), the average size of nickel grain in NS-1.8 composites was estimated to 0.91 μm, whereas that of NS-3.2 composites was 0.43 μm. However, the average size of nickel grain in NS-2.5 composites was only 0.12 μm. The results were proved by SEM images in Figure 2 and the conclusion proposed by Khuram et al. [30].

### 3.4. Corrosion Behaviour Test

**Figure 6:**
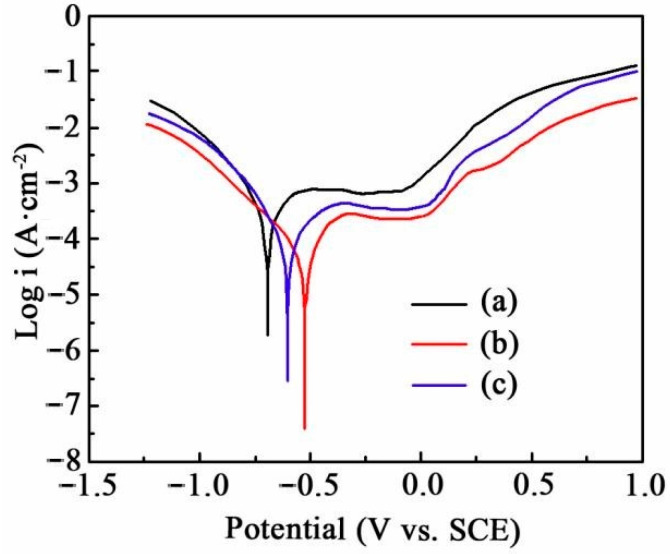
Potentiodynamic polarization curves of Ni-SiC composites manufactured at various current densities: (a) 1.8 A/dm^2^, (b) 2.5 A/dm^2^, and (c) 3.2 A/dm^2^.

The potentiodynamic polarization curves of Ni-SiC composites immersed in the corrosion medium are revealed in Figure 6. Generally, the current density and corrosion potential of composites was highly related to the microstructure [31]. The corrosion current density of Ni-SiC composites decreased firstly and then increased with the growth of current density. The corrosion current density of NS-1.8, NS-2.5 and NS-3.2 composites were 4.718, 2.439, and 3.956 μA/cm^2^, respectively. In addition, the corrosion potentials of NS-1.8, NS-2.5, and NS-3.2 composites were −0.732, −0.507, and −0.618 V, respectively. Li et al. [32] proposed that the small corrosion current density and high corrosion potential of Ni-based composites owned the excellent anti-corrosion ability. Therefore, the corrosion resistance of NS-2.5 composites was better than those of NS-1.8 and NS-3.2 composites.

**Figure 7:**
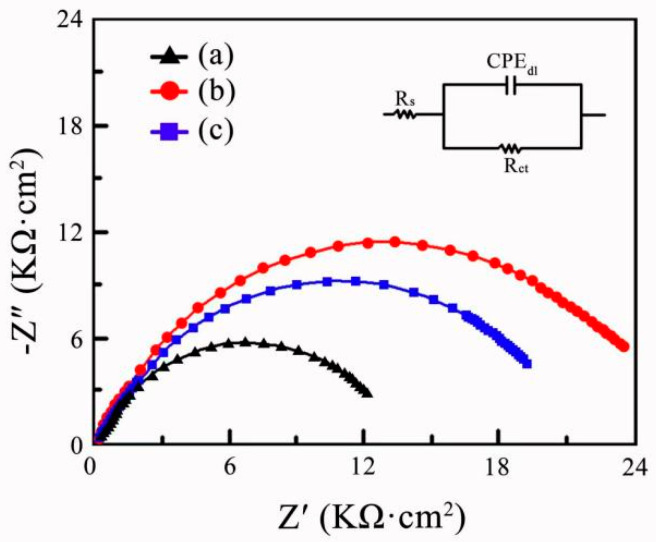
Nyquist plots and equivalent circuit graph of Ni-SiC composites prepared at different current densities: (a) 1.8 A/dm^2^, (b) 2.5 A/dm^2^, and (c) 3.2 A/dm^2^.

The Nyquist plots and equivalent circuit diagram of Ni-SiC composites immersed in the corrosion medium are revealed in Figure 7. The anti-corrosion performance of nicke-based composites was closely relevant to the impedance value (Z) of composites, which has been demonstrated by the research of Li et al. [33]. As impedance value increased, the resistance of charge conduction rose. After the corrosion current density reduced, the corrosion resistance of composites enhanced. By contrast, the impedance value of NS-1.8 composites was lower than those of NS-2.5 and NS-3.2 composites, demonstrating the anti-corrosion ability of NS-1.8 composites was terrible. Besides, the impedance value of Ni-SiC composites rose with the growth of current density. The impedance value of NS-2.5 composites was nearly 2 times larger than that of NS-1.8 composites. However, in comparison to NS-3.2 composites, the impedance value of NS-1.8 composites was approximately reduced by half, resulting in the significant reduction of anti-corrosion performance.

**Figure 8:**
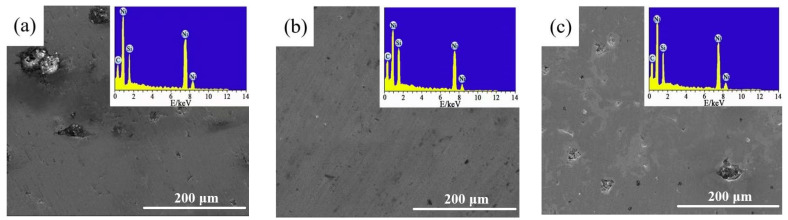
Corrosion morphologies and EDS results of Ni-SiC composites produced at various current densities: (**a**) 1.8 A/dm^2^, (**b**) 2.5 A/dm^2^, and (**c**) 3.2 A/dm^2^.

Figure 8 exhibits the corrosion morphologies and EDS results of Ni-SiC composites produced at various current densities. The observed SEM graphs revealed that the small and shallow corrosion pits appeared on the surface of NS-2.5 composites, while the large and deep corrosion pits emerged on the surfaces of NS-1.8 and NS-3.2 composites. In addition, the EDS results of three Ni-SiC composites demonstrated that the Fe element was not discovered, which illustrated that the corrosion medium was not contacted with Q235 steel substrate and generated corrosion products. The reason of these results could be explained as: (1) The NS-2.5 composites owned a compact and flatted structure, which could obviously hinder the corrosion medium of NaCl and Na_2_SO_4_ attack, leading to the outstanding anti-corrosion ability [34]. Meanwhile, the corrosion resistance of SiC nanoparticle was better than that of nickel, leading to the NS-2.5 composites with high SiC content owned excellent anti-corrosion performance. Furthermore, the uniform distribution of SiC nanoparticles in the composites could greatly extend the corrosion path and improve the corrosion resistance of composites. The conclusion has been confirmed to the study reported by Hong et al. [35].

**Figure 9:**
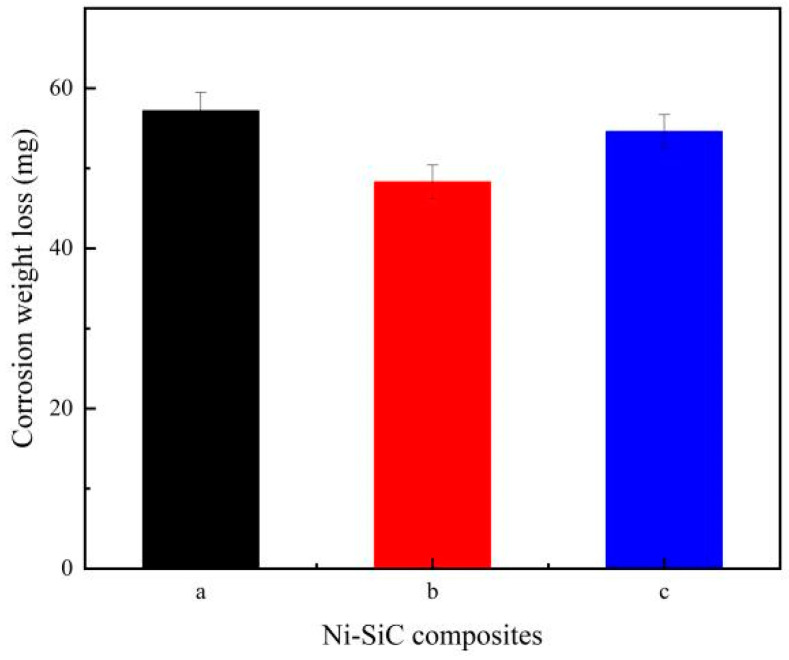
Corrosion weight loss of Ni-SiC composites deposited at different current densities: (a) 1.8 A/dm^2^, (b) 2.5 A/dm^2^, and (c) 3.2 A/dm^2^.

Figure 9 presents the corrosion weight loss of Ni-SiC composites immersed in the corrosion medium. The measured outcome showed that the corrosion weight loss of Ni-SiC composites decreased firstly and then increased with the growth of current density. The corrosion weight losses of NS-1.8, NS-2.5, and NS-3.2 composites were 57.2, 48.3, and 54.6 mg. The causes of the phenomenon could be attributed to the SiC content and micro-structure of composites. Nujira et al. [36] discovered that the anti-corrosion ability of nanoparticles reinforced phase was superior to that of nickel matrix. Furthermore, the loose and uneven micro-structure of Ni-SiC composites could not effectively hinder the damage of corrosion medium [37]. Hence, the corrosion weight loss of NS-2.5 composites was lower than those of NS-1.8 and NS-3.2 composites.

### 3.5. Corrosion Relationship Analysis

**Figure 10:**
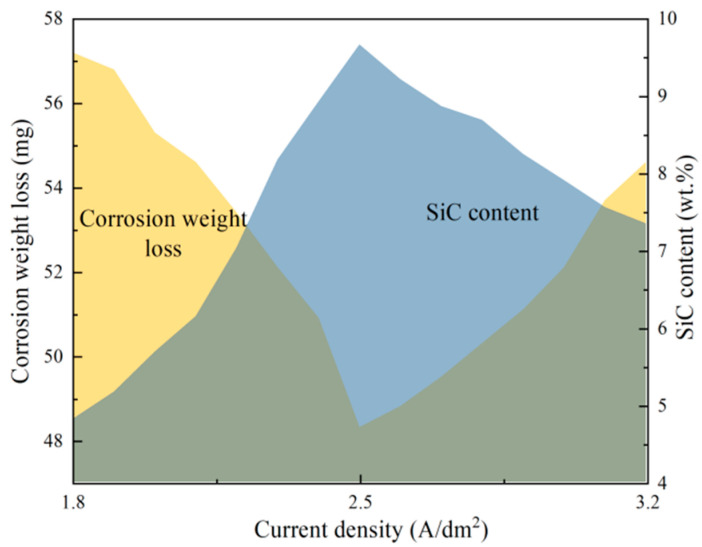
Corrosion relationship between SiC content and corrosion weight loss of Ni-SiC composites fabricated at various current densities.

The corrosion relationship of current density on the SiC content and corrosion weight loss of Ni-SiC composites are demonstrated in Figure 10. On the one hand, the SiC content of Ni-SiC composites increased firstly and then decreased with the growth of current density. On the other hand, the corrosion weight loss of Ni-SiC composites decreased initially and then increased with the growth of current density. The SiC content and corrosion weight loss of Ni-SiC composites fabricated at current density of 2.5 A/dm^2^ were the highest of 9.67 wt.% and the lowest of 48.3 mg, respectively. The reason of this phenomenon could be illustrated that the feasible current density contributed to the deposition of SiC nanoparticles and the formation of dense and flatted structure, resulting in the corrosion weight loss of composites reduced and the anti-corrosion performance improved [38].

## 4. Conclusions

The Ni-SiC composites were successfully prepared using electrodeposition approach. The present study emphasized the significant influence of current density on the surface morphology, phase structure and anti-corrosion performance of Ni-SiC composites. This could provide experimental data for anti-corrosion ability of Ni-SiC composites in the storage tank application and other Ni-based composites. The following conclusions could be drawn:

1. The Ni-SiC composites manufactured at 1.8 or 3.2 A/dm^2^ had uneven and loose microstructures, while the Ni-SiC composites fabricated at 2.5 A/dm^2^ possessed smooth and compact microstructure. In addition, EDS results shown that the SiC content of Ni-SiC composites prepared at 1.8 or 3.2 A/dm^2^ were lower than the one obtained at 2.5 A/dm^2^.
2. XRD patterns demonstrated that the SiC phases were successfully incorporated into the Ni matrix during electrodeposition. Furthermore, the average size of nickel grain in the Ni-SiC composites produced at 2.5 A/dm^2^ was smaller than those deposited at 1.8 or 3.2 A/dm^2^.
3. Electrochemical measurement indicated that the Ni-SiC composites fabricated at 2.5 A/dm^2^ owned a low corrosion current density, positive corrosion potential, and large impedance value, illustrating the corrosion resistance was excellent. Moreover, the corrosion relationship revealed that the corrosion weight loss of Ni-SiC composites was highly related to SiC content.

## Figures and Tables

**Table 1:** Electrolyte composition and Plating parameters for fabricating Ni-SiC composites.

Electrolyte Composition	Concentration (g/L)
SiC concentration	8
NiSO_4_·6H_2_O	230
C_6_H_8_O_7_·H_2_O	30
NiCl_2_·6H_2_O	40
H_3_BO_3_	35
Saccharin	4
**Plating Parameters**	**Specific**
Duty cycle (%)	60
Plating temperature (°C)	55
Plating time (min)	45
pH value	4.1
Rational speed (rad/min)	600
Frequency (Hz)	10

## Data Availability

Data is contained within the article.
